# Supplementary material for: “If he returns, receive him because he has realized that he needs assistance”: A qualitative study exploring preferences for retention on antiretroviral therapy support in Malawi among Lighthouse Clinic clients
Source: PLoS One. 2025 May 27;20(5):e0324273. doi: 10.1371/journal.pone.0324273 (PMC12112084; doi:10.1371/journal.pone.0324273)
Supplement: Attachment — Submitted filename: Comments to the authors-PLOS One.docx [file pone.0324273.s001.docx]

Comments to the Author

The qualitative review addresses an important topic and is mostly clearly described. However, some areas can be improved for readers to grasp the information more clearly.

Major comments:

1. The title is very long but lacks the necessary information. A good title should present the major area, study participants, site and the study design.
2. The article's introduction is lengthy; a more concise version would be helpful since the details are repeated in the methods section.
3. Lines 18 and 54: This manuscript focuses on retention in HIV care, but the term "retention in ART" is used in some places. The authors should consistently use "retention in HIV care," which accurately reflects the phenomenon.

Minor comments:

1. Consider revising the headings, such as instead of materials and methods only using methods.
2. Consider making the writing more succinct by removing redundant sentences.
3. Line 22. Objectives are unclear as they don’t elaborate on exploring mHealth intervention. The objectives should state that we aimed to explore participants' experiences for improved retention in care using 2-way texting retention support.
4. Line 19-Line 20 “Retention is not static---gaining traction can be removed.
5. Line 26,” on ART,” is incomplete; it should read like on retention in care.
6. Line 29,” We used the Re-Aim should be before Line 28,” Rapid qualitative.”
7. Results: Lines 31-39 are like the conclusion; results should summarize the themes and subthemes from which conclusions can be drawn.
8. Introduction. Line 56. Consider using the terms adherence to ART and retention in HIV.
9. Discussion: Line 473-487. This section requires organization and is more focused on summarizing what was explored and what was found. The framework should be discussed earlier, not in the last sentence.
10. Conclusion: Consider making this section, more relatable to the study. Line 575 -Line 580 can be in the future directions.
11. References: all websites cited should also have a date of access.
