# Supplementary material for: “If he returns, receive him because he has realized that he needs assistance”: A qualitative study exploring preferences for retention on antiretroviral therapy support in Malawi among Lighthouse Clinic clients
Source: PLoS One. 2025 May 27;20(5):e0324273. doi: 10.1371/journal.pone.0324273 (PMC12112084; doi:10.1371/journal.pone.0324273)
Supplement: Attachment — Submitted filename: Response to Reviewers.docx [file pone.0324273.s004.docx]

13 January 2025

Editorial Team, PLOS ONE,

Dear Joseph KB Matovu:

Thank you for your email regarding our manuscript entitled, “’If he returns, receive him because he has realized that he needs assistance’: exploring cyclical retention with antiretroviral therapy clients at Lighthouse Clinic, Malawi.” We appreciate the thoughtful comments and suggestions from you and the reviewers and the invitation to submit a revised manuscript.

We have addressed the concerns in detail below and are pleased to re-submit this revised version for your consideration.

Please do not hesitate to contact me if we can clarify any points.

Sincerely,

Gillian O’Bryan, MPH

International Training and Education Center for Health (I-TECH)

Department of Global Health

Schools of Medicine and Public Health & Community Medicine

University of Washington

1. Major comments

| **No.** | **Area of concern** | **Reviewer’s comment** | **Author’s response** |
| --- | --- | --- | --- |
| 1 | Title | The title is very long but lacks the necessary information. A good title should present the major area, study participants, site, and study design. | Thank you for this suggestion, we’ve included additional detail in the title to better reflect design and major area of study. |
| 2 | Introduction | The article’s introduction is lengthy; a more concise version would be helpful since the details are repeated in the methods section. | Thank you for this suggestion. We’ve reworked the introduction to be more concise and less duplicative of the methods section. |
| 3 | Abstract/Introduction | Lines 18 and 54: This manuscript focuses on retention in HIV care, but the term “retention in ART” is used in some places. The authors should consistently use “retention in HIV care,” which accurately reflects the phenomenon. | Thank you for this suggestion. While retention in HIV care is more encompassing, it is retention on ART that we are interested in as a key and well known indicator of HIV treatment success. ART retention is critical for achieving viral suppression. We therefore prefer to keep this terminology, see also (https://www.who.int/teams/global-hiv-hepatitis-and-stis-programmes/hiv/treatment/service-delivery-adherence-retention#:~:text=building%20and%20mentoring.-,Adherence%20and%20retention,as%20well%20as%20treatment%20monitoring.). Additionally, retention on ART is the preferred term used by the Lighthouse Trust. As such, we have not changed retention on ART to retention in HIV care throughout the manuscript but did mention HIV care where we felt this was clearer. |

2. Minor comments

| **No.** | **Area of concern** | **Reviewer’s comment** | **Author’s response** |
| --- | --- | --- | --- |
| 1 | Headings | Consider revising the headings, such as instead of materials and methods only using methods. | Thank you for this suggestion, we used manuscript organization guidance outlined on the PLOS ONE website which includes “Materials and Methods” as a heading (<https://journals.plos.org/plosone/s/submission-guidelines#loc-manuscript-organization>). |
| 2 | General | Consider making the writing more succinct by removing redundant sentences. | Thank you for this feedback. We’ve reorganized the introduction and discussion sections to make the writing more succinct. |
| 3 | Abstract-Introduction | Line 22, objectives are unclear as they don’t elaborate on exploring mHealth intervention. The objectives should state that we aimed to explore participants’ experiences for improved retention in care using 2-way texting retention support. | Thank you for this suggestion, we’ve revised the objective statement in the abstract. |
| 4 | Abstract-Introduction | Line 19 – 20, “Retention is not static—gaining traction can be removed. | Thank you for this suggestion, we’ve revised the abstract but kept sentences around mHealth and retention over time as they are key providing background on the study’s objectives. |
| 5 | Abstract-Methods and Materials | Line 26, “on ART,” is incomplete; it should read like on retention in care. | See 1.3 above. |
| 6 | Abstract-Methods and Materials | Line 29, “We used the Re-Aim” should be before line 28, “Rapid qualitative.” | Thank you for this suggestion, we have incorporated this change into the revised version of the manuscript. |
| 7 | Abstract-Results | Lines 31 – 39 are like the conclusion; results should summarize the themes and subtheme from which conclusions can be drawn. | Thank you for this suggestion. We’ve reorganized the abstract with results summarizing main themes. |
| 8 | Introduction | Line 56. Consider using the terms adherence to ART and retention in HIV. | See 1.3 above. |
| 9 | Discussion | Line 473-487. This section requires organization and is more focused on summarizing what was explored and what was found. The framework should be discussed earlier, not in the last sentence. | Thank you for this suggestion, we’ve reorganized and shortened the discussion section as well as included the RE-AIM framework earlier. |
| 10 | Strengths and limitations | Consider making this section more relatable to the study. Line 575 – line 580 can be in the future directions. | Thank you for this suggestion, we’ve rewritten the conclusions to be more relatable to the study. |
| 11 | References | All websites cited should also have a date of access. | Thank you for identifying this, all cited websites now have a date of access. |
