# Supplementary material for: “If he returns, receive him because he has realized that he needs assistance”: A qualitative study exploring preferences for retention on antiretroviral therapy support in Malawi among Lighthouse Clinic clients
Source: PLoS One. 2025 May 27;20(5):e0324273. doi: 10.1371/journal.pone.0324273 (PMC12112084; doi:10.1371/journal.pone.0324273)
Supplement: Attachment — Submitted filename: Reviewers comments.docx [file pone.0324273.s003.docx]

If he returns, receive him because he has realized that he needs assistance”: a qualitative study exploring preferences for retention on antiretroviral therapy support in Malawi among Lighthouse Clinic clients

The authors explored the perspectives of people living with HIV on retention support, including a two-way texting (2wT) mHealth intervention, in two Malawian clinics.

They have addressed all queries and revised the manuscript accordingly.
