# Supplementary material for: “If he returns, receive him because he has realized that he needs assistance”: A qualitative study exploring preferences for retention on antiretroviral therapy support in Malawi among Lighthouse Clinic clients
Source: PLoS One. 2025 May 27;20(5):e0324273. doi: 10.1371/journal.pone.0324273 (PMC12112084; doi:10.1371/journal.pone.0324273)
Supplement: Attachment — Submitted filename: Response_to_Reviewers_auresp_2.docx [file pone.0324273.s005.docx]

19 March 2025

Editorial Team, PLOS ONE,

Dear Joseph KB Matovu:

Thank you for your email regarding our manuscript entitled, “’If he returns, receive him because he has realized that he needs assistance’: exploring cyclical retention with antiretroviral therapy clients at Lighthouse Clinic, Malawi.” We appreciate the thoughtful comments and suggestions from you and the reviewers and the invitation to submit a revised manuscript.

We have addressed the concerns in detail below and are pleased to re-submit this revised version for your consideration.

Please do not hesitate to contact me if we can clarify any points.

Sincerely,

Gillian O’Bryan, MPH

International Training and Education Center for Health (I-TECH)

Department of Global Health

Schools of Medicine and Public Health & Community Medicine

University of Washington

1. Review Comments to Author

| **No.** | **Area of concern** | **Reviewer’s comment** | **Author’s response** |
| --- | --- | --- | --- |
| 1 | Introduction | Line 75-76: It is not clear if there is no latest data available to talk to the LTFU rates in Malawi apart from the 33% in 2015. It will be good to have the latest data. | Thank you for this suggestion, we’ve revised the sentence with a 2022 updated estimate for retention on ART at 12 months after initiation. |
| 2 | Results | Just to note: the table under the results section line 192, still does not have a title/heading. | Thank you for identifying this omission, a title has been added to the table (table 2) in the results section. |
